# “Disability Grief”: A patient’s allegorical expression of her disability

**Published:** 2019-10-07

**Authors:** Abdorreza Naser Moghadasi

**Affiliations:** Multiple Sclerosis Research Center, Neuroscience Institute, Tehran University of Medical Sciences, Tehran, Iran

**Keywords:** Multiple Sclerosis, Grief, Disability

Multiple sclerosis (MS) is an autoimmune disabling disease of the central nervous system, and can lead to a wide range of symptoms. Although the most common form of MS is relapsing-remitting, most of the patients also will develop disability in the future due to the natural course of the disease.^1^^,^^2^ The disease onset mostly occurs in 27 years of age.^3^ Therefore, considering the symptoms and complications of the disease, it can virtually affect the whole life of the patient. Unfortunately, there is not any accurate study of the opinions of patients with MS regarding their illness. However, in the daily work of treatment, one of the well-known cases of severe discomfort expressed by patients is the fear of future disability. This issue is aggravated when a person starts to lose abilities. When the patient experiences disability in walking and a decrease in his/her daily activities, thinking about the future becomes one of his/her major concerns.

Most patients express these concerns in their visits. However, a patient who has other abilities such as poetry, writing a story, or painting, can more effectively portray concerns.

The painting reported here is the allegorical view of a patient about her disability.

The patient is a 37-year-old woman who has had MS for 15 years. Her illness started with the right eye optic neuritis. She has had six attacks during this period and, after 9 years, her disease entered the secondary progressive phase. Her symptom in this stage was the weakness of the right lower extremity, which was gradually deteriorating such as difficulty in walking. Now, she is unable to walk without help. This disability has affected all aspects of her life, and has greatly diminished her quality of life. She has been interested in drawing since childhood, and has drawn as a non-professional artist. One of her paintings reported here (Figure 1), according to the patient herself, reflects her perception and grief over her progressive disability. She believes that she should say goodbye to her ability and good days of the past. She painted herself as someone (when she could walk unrestrictedly) moving away along with a balloon.

**Figure 1:**
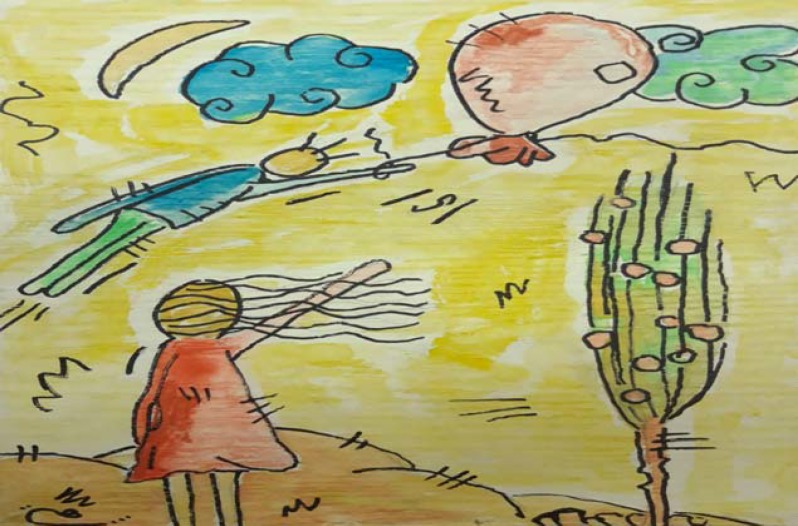
“Disability Grief”. She painted herself as someone (when she could walk unrestrictedly) moving away along with a balloon

This is while she herself (with the disability that she already has) is standing somewhere on the ground saying goodbye to her past (with regret) which is flying and moving away. 

As a physician who treats patients with MS, I believe that this painting is a picturesque and, at the same time, the most pathetic conceptions of the patients about themselves and their lives.

Disability is a main core of MS. Although most of patients have relapsing-remitting course, many of them have residual impairment after each relapse; and this impairment can cause disability, and influence their lives. So, disability can reduce the quality of life. All of these may cause grief. The patient remembers his/her previous life, when he/she had not disability. The physicians should consider this grief. They must calm the patients, and help them to manage the new situation.

## Notes:

**How to cite this article:** Naser Moghadasi A. “Disability Grief”: A patient’s allegorical expression of her disability. Iran J Neurol 2019; 18(4): 190-1.

## References

[1] Lorscheider J, Buzzard K, Jokubaitis V, Spelman T, Havrdova E, Horakova D (2016). Defining secondary progressive multiple sclerosis. Brain.

[2] Rezaali S, Khalilnezhad A, Naser MA, Chaibakhsh S, Sahraian MA (2013). Epidemiology of multiple sclerosis in Qom: Demographic study in Iran. Iran J Neurol.

[3] Sahraian MA, Khorramnia S, Ebrahim MM, Moinfar Z, Lotfi J, Pakdaman H (2010). Multiple sclerosis in Iran: A demographic study of 8,000 patients and changes over time. Eur Neurol.

